# PUBLIC POLICY ISSUES OF SIGNIFICANCE TO THE BIOLOGICAL SCIENCES

**DOI:** 10.1093/geroni/igz038.1454

**Published:** 2019-11-08

**Authors:** Scott Leiser

**Affiliations:** University of Michigan, Ann Arbor, Michigan, United States

## Abstract

This presentation will cover public policy issues of significance to the aging population, focusing on the perspective of the biological sciences and on policies that may improve physical and mental health.

